# A biphasic navigational strategy in loggerhead sea turtles

**DOI:** 10.1038/s41598-020-75183-6

**Published:** 2020-10-22

**Authors:** Paolo Luschi, Dogan Sözbilen, Giulia Cerritelli, Franck Ruffier, Eyup Başkale, Paolo Casale

**Affiliations:** 1 Department of Biology, University of Pisa, Pisa, Italy; 2 Department of Veterinary, Acıpayam Vocational School, Pamukkale University, Denizli, Turkey; 3 Aix Marseille University, CNRS, ISM, Marseille, France; 4 Department of Biology, Faculty of Arts and Sciences, Pamukkale University, Denizli, Turkey

**Keywords:** Animal migration, Animal behaviour

## Abstract

The homing journeys of nine loggerhead turtles translocated from their nesting beach to offshore release sites, were reconstructed through Argos and GPS telemetry while their water-related orientation was simultaneously recorded at high temporal resolution by multi-sensor data loggers featuring a three-axis magnetic sensor. All turtles managed to return to the nesting beach area, although with indirect routes encompassing an initial straight leg not precisely oriented towards home, and a successive homebound segment carried out along the coast. Logger data revealed that, after an initial period of disorientation, turtles were able to precisely maintain a consistent direction for several hours while moving in the open sea, even during night-time. Their water-related headings were in accordance with the orientation of the resulting route, showing little or no effect of current drift. This study reveals a biphasic homing strategy of displaced turtles involving an initial orientation weakly related to home and a successive shift to coastal navigation, which is in line with the modern conceptual framework of animal migratory navigation as deriving from sequential mechanisms acting at different spatial scales.

## Introduction

Understanding the orientation and navigation systems of migrating animals is a most relevant, but still unsolved, issue in behavioural biology^1^. The scientific study of these phenomena is faced with a variety of technical and logistical problems, mostly due to the difficulties in experimenting with wild animals freely moving in their environment or in reliably tracking them as they roam over hundreds of km. This is especially true for the many migrations that take place in the oceanic environment, often far from coast, where the challenges to study navigational abilities are further exacerbated^2^. A most useful approach to study animal navigation is to perform the so-called displacement experiments, in which an animal is moved from a familiar site to a novel location, tens or hundreds of km away^2–4^. Experimental translocations of this kind pose a great challenge to marine animals, especially when released offshore, as they find themselves in a previously unknown location and have then to navigate in the seemingly featureless oceanic environment. Yet, several studies have shown how sea turtles and seabirds are typically able to correct for displacement and to get back to the original capture site^5,6^ or to other locations that are meaningful for them (for example a migratory corridor^7^), often showing oriented responses even soon after release^8^.

Marine turtles are well-known examples of oceanic navigators, as they are able to perform long-distance oceanic migrations navigating towards specific destinations like a nesting beach or a foraging area. The navigational mechanisms allowing such feats are however mostly unknown, and only in recent years the issue has been investigated directly thanks to specific experimental studies, often involving satellite telemetry techniques^2^. Only a limited number of displacement experiments has been carried out on sea turtles, also given the non-negligible logistical problems associated with translocating such large animals for tens of km^2,9^. These studies have documented the homing abilities in natural conditions of green (*Chelonia mydas*) and loggerhead (*Caretta caretta*) turtles^5,10–16^, in accordance with findings obtained under more controlled conditions involving virtual magnetic displacements^17^. Physically displaced turtles have indeed been able to return to the home locations even after displacements of hundreds of km in the open sea, and so are thought to be able to determine their position with respect to the home location, likely through a combination of different navigational mechanisms such as beaconing, pilotage and true navigation^18,19^. These studies have however shown that turtles often follow indirect routes to home, taking curved or circuitous paths and/or making detours in their homing trip, that are largely different from the straight homing paths recorded after displacement in birds^18^. This phenomenon is particularly evident after long-distance (> 100 km) offshore translocations of turtles nesting in isolated oceanic islands, where the navigational challenge of returning home after the experimental treatment is most relevant: in these cases turtles often return home only after days or weeks of tortuous and seemingly fruitless movements, that have been interpreted as searches for the home island^5,14,15,20^, but whose nature is still unclear.

The navigational abilities of displaced turtles have so far been investigated relying only on the location data provided by satellites, assessing the orientational responses of displaced turtles on the basis of features of the reconstructed movement paths, such as their overall length or tortuosity. However, such an approach can only provide a rough indication of the actual turtle orientation, as the satellite-derived paths derive from the summed effect of the turtles’ active movement and of the drift of oceanic currents, which can be substantial in some cases leading to path that are curved or not directed towards destination^21,22^. While the role of current drift along the turtles’ routes can be estimated through oceanographic models, so to evaluate the current-corrected orientation of swimming turtles^14,15,23^, this indirect procedure is somewhat imprecise, being affected by the accuracy of tracking data and by the reliability of modelled currents^22,24^. Also, information on turtle orientation is generally available with a limited temporal accuracy (several hours at least) being dependent on the low and variable frequency of satellite locations. The only complete and unbiased way to assess the turtles’ orientational responses is to directly measure the turtles’ actual orientation with respect to the geomagnetic field, employing instruments featuring a magnetic sensor by which the actual (water-related) magnetic headings of instrumented animals can be monitored^25,26^, even at very high frequencies (typically, seconds). Unfortunately, such devices are only available as data loggers that have to be retrieved to obtain stored information: as such, they have been used on marine turtles only in special situations (for example^25,27,28^), and in no case to investigate turtle orientation after displacement.

In the present study, we have overcome these shortcomings by employing multi-sensor data loggers along with satellite transmitters. This allowed us to track the movements of displaced turtles by satellite while simultaneously recording the turtles’ magnetic headings during their homing trip, so to closely correlate the movements made by the turtles with the underlying orientation and navigation processes. With these experiments we also aimed at investigating further the navigational abilities of marine turtles, by studying for the first time the homing abilities of turtles living in the Mediterranean Sea, that are known to perform long-distance movements at all stages of their life cycle^29^. These turtles move inside a semi-enclosed basin rich in coastlines and islands, where the available orientation and navigational cues likely differ from those used for movements in the oceanic environment that have been mostly explored in previous experiments. More specifically, we aimed to (i) assess whether turtles displaced in an area where multiple potential nesting sites exist, still display fidelity to the original beach or shift to another location; (ii*)* test whether turtles displaced not far away from a continental coast display a biphasic navigational strategy first reaching the nearest coast and then following it towards the final destination; (iii*)* determine if they stop along the route in non-nesting coastal habitats before reaching the final destination, so to estimate their ‘urgency’ in reaching the home area; (iv*)* evaluate the orientation efficiency during the different path segment(s) in terms of short-term directionality along the route, so to assess the effect of deviating factors that may produce any indirect path.

## Results

**Figure 1:**
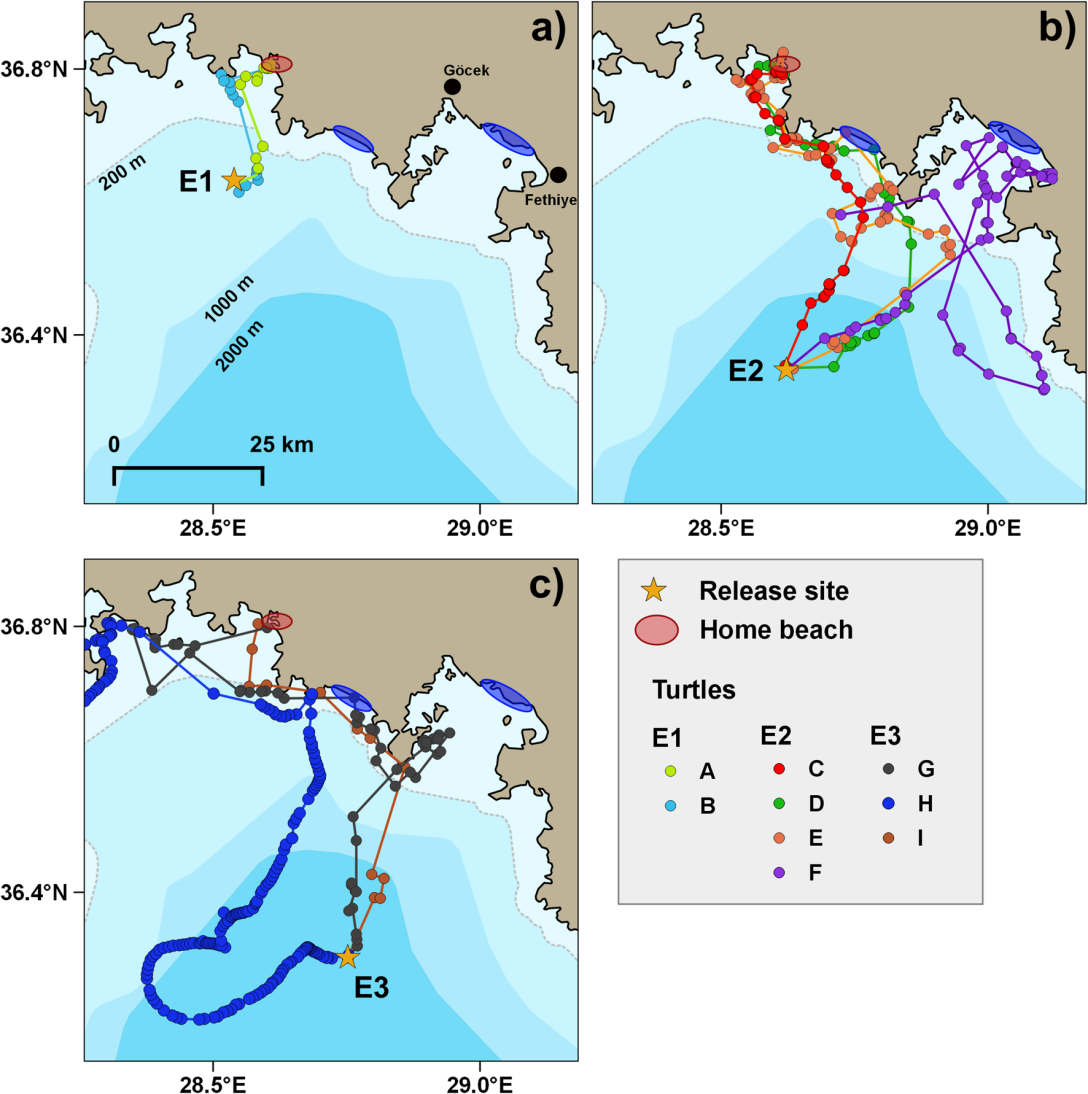
Homing routes of turtles released in **(a)** experiments E1, **(b)** experiment E2 and **(c)** experiment E3. The blue ellipses show the location of other known loggerhead nesting areas in the region. Turtles H and I did not make another nesting attempt after completing the homing movement, while turtle F remained for more than 10 days in Fethiye bay without nesting, before returning to Iztuzu beach. The map was created on QGIS 3.12.2 (https://www.qgis.org). The bathymetry data was added using Natural Earth free vector and raster map data (naturalearthdata.com).

Overall, all displaced turtles managed to compensate for the displacement and to return at least to the general vicinity of the nesting beach (Fig. 1a,b). However, they mostly followed quite indirect routes, as shown by homing route straightness indexes that were below 0.55 in 7 cases out of 9 and averaged to 0.49 (n = 9, median 0.47; range 0.76–0.22; Supplementary material Table S1), which means that their path was on average nearly twice as long as the shortest one.

This lack of homeward directedness was primarily due to fact that the first part of the track was not directed towards the nesting beach. The two turtles of experiment E1 and three of experiment E2 (Fig. 1a) displayed a similar north-east movement after release, that was quite short in E1 turtles and much more prolonged in the other cases. Turtles C (Fig. 1a), I and G (Fig. 1b) followed a broadly similar northward course, while turtle H (Fig. 1b) moved westward for about 8 h after release, and then spent the following two days making a large loop to the west of the release site. Only after 48 h from release, did she started to keep a northward route that led her to reach the coast about 4.4 days after release.

### Open sea orientation

**Figure 2:**
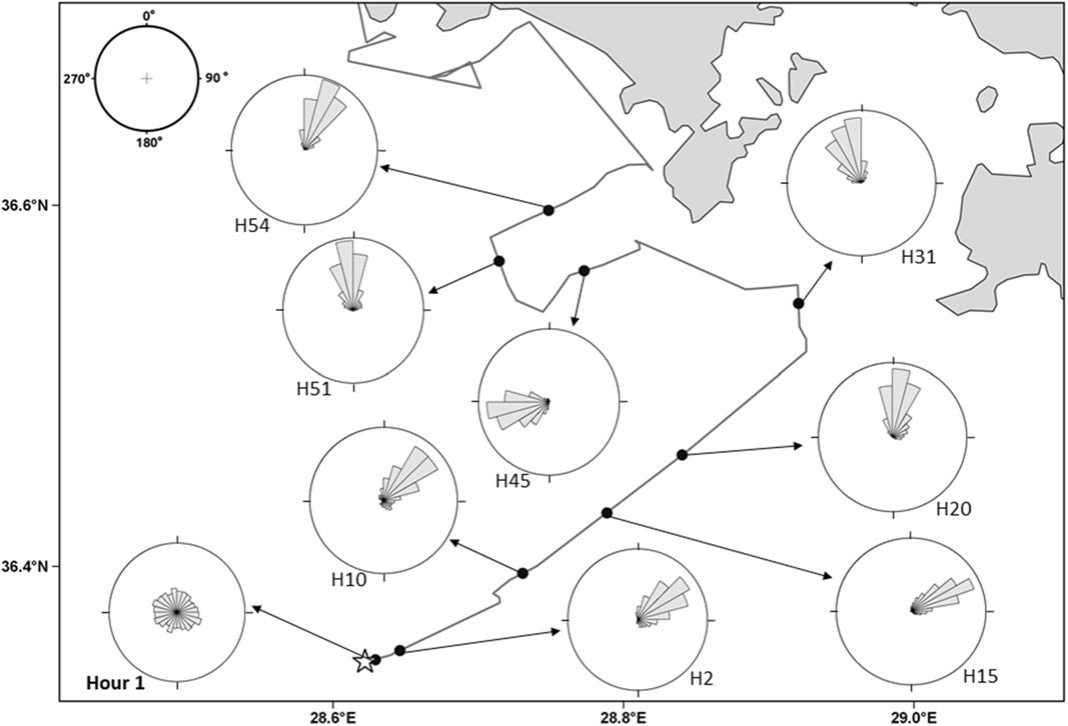
Distributions of magnetic headings recorded in hourly samples along turtle’s E route. The star indicates the release site.

**Figure 3:**
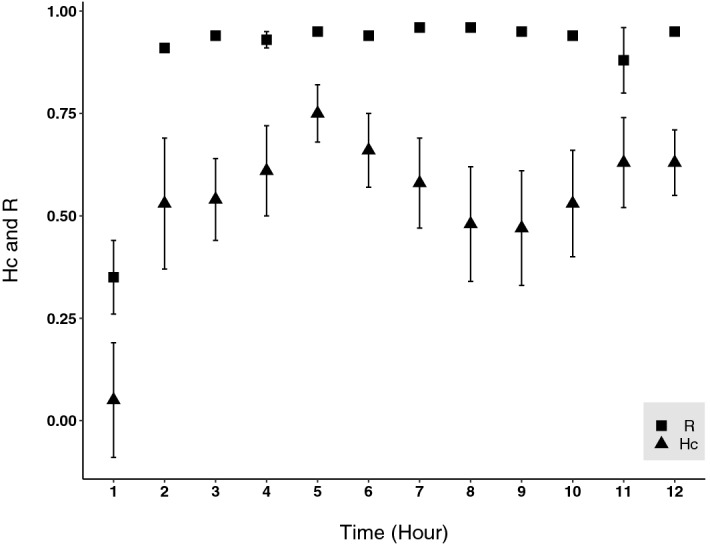
Concentration of headings and orientation towards home of the turtles during the first 12 h after release. Data shown are mean + /− SEM of vector length (R, squares) and homeward component (Hc, triangles) averaged for the six turtles calculated for hourly samples of magnetic headings (3600 headings each). Homeward components of the same samples express the turtle tendency to orient towards the home direction. Home direction for successive samples was computed by measuring the directions of hourly-interpolated Argos locations.

The heading data recorded for these turtles were largely in accordance with the orientation of their satellite-reconstructed path. To assess this, we calculated the mean vectors for the distribution of directions between successive interpolated locations and for the actual heading data recorded by the logger. To standardise the procedure among different turtles, we computed these values for the first 13 h after release, excluding the first hour where turtles did not show oriented behaviour (see below). The vectors for each individual turtle have an average difference of 8° in orientation (range: 2°–12°) and of 0.04 in length (range: 0.01–0.08), revealing a close similarity between the actual orientation of the turtle and that of the reconstructed track. Heading data also revealed that the six monitored turtles oriented in variable directions in the first hour after release, then shifting to a highly oriented behaviour afterwards (example in Fig. 2). Their hourly distributions of headings were indeed concentrated around a given direction for the successive 11 h at least (Fig. 3), with the directions chosen in this period being often similar in the different turtles but poorly oriented towards home (Fig. 3, Supplementary material Fig. S1). As a result, the distribution of mean vectors of the different turtles in the first hour was not different from random (Hotelling test, n = 6, P = 0.52, Supplementary material Fig. S1) and was always significantly oriented towards Northeast in the successive 1-h periods (Hotelling tests, n = 6, always P < 0.001; Supplementary material Fig. S1). These distributions were however often not directed towards home, as shown by the low values of homeward component recorded in the presence of vector lengths close to 1 (Fig. 3).

Course corrections en route were also evident in the heading data, for instance in turtles D, C, and E (Fig. 2) that changed their orientation while still far offshore. In all cases, turtles were able to maintain their orientation during the night, as the mean vector distribution of the diurnal segments was not significantly different from the nocturnal ones (Hotelling test: n = 5, T^2^ = 21.71, P = 0.07).

### Coastal movements

These initial movements led turtles to reach sooner or later the coast, from which they generally oriented towards home mostly following the coastline. The turtles of the first release made landfall on a rocky peninsula about 8 km west of the beach, from where they quickly reached their final destination. The turtles of the other (more distant) releases first reached the Turkish coastline, mostly at the gulf of Fethiye mouth, about 35 km Southeast of the home beach (Fig. 1a,b), an area that hosts some loggerhead nesting beaches (Fig. 1). From there, turtles moved quite directly towards the home beach, with the exception of turtle F, that entered the gulf and remained in the bay of Fethiye for about 10 days. During this period, she did not spend any long period out of water (> 10 min), as assessed from the logger’s depth records (data not shown), so we can exclude that she nested in the area. The turtle then moved away from the gulf on 8 June, making a large offshore loop before returning to the mouth of the gulf after about 46 h. Contacts with the transmitter were then lost on 10 June, when the turtle was apparently moving offshore again. She was then found nesting again at Iztuzu in the night of 21 June, 29 days after her previous nesting, having lost the Argos unit but not the logger. Depth data stored in the logger revealed that the turtle spent three periods of at least 60 min out of the water during the night of 10–11 June, having likely nested on a beach east of Iztuzu, an area where turtle nesting is known to occur^30,31^.

After reaching the coast, the other turtles continued to move decidedly towards the nesting beach, without stopping for a significant amount time at any site along the coast. Their movement speed during this inshore leg of the journey was similar to that of the previous offshore segment and their headings continued to be oriented in a given direction (Figure S2). Only after having reached the waters off the nesting beach, did they slow down and ceased to orient in a given direction (Supplementary material Fig. S2).

### Final approach to home and successive movements

Turtles of releases E2 and E3 had a similar final approach to home (Supplementary material Fig. S3), as they all left the coast when they were about 10 km south of the beach, crossed the Iztuzu bay and arrived close to the same peninsula reached by the two turtles of experiment E1. This behaviour was evident especially for turtles C, E and D but was likely displayed by turtle I as well, for which however few localizations were obtained. Turtles G and H too passed from the coastal area south of the home beach, but then moved further offshore than the other turtles and reached the bay of Marmaris, about 25 km west of Iztuzu beach. Turtle G then moved east and finally reached its destination, while turtle H moved south and then southwest towards the open sea. It is worth noting that turtle G visited the Marmaris bay area again after having stayed for 4 days in front of Iztuzu and before returning there to nest. Turtle H conversely never returned to the Iztuzu area, and rather reached the northern coast of Crete where she was then localised for the following months (data not shown), having reached her foraging grounds.

After completing their homing journey, all turtles were localised in the waters immediately in front of the beach and inside the nearby lagoon for 8–12 days. The only exception to this pattern was represented by turtles H and I, that left the area two days after completing homing. The other turtles were then found nesting again on Iztuzu beach 14–16 days after their initial capture (turtle F after 29 days), except for turtle B who was not seen nesting again before she left the area on 9 July. Since she moved away from the area 14 days after her capture, we think that she may have nested another time before leaving, although she was not found while nesting and no indications were collected either from Argos locations or satellite-relayed sensor data as to whether she had nested or not in this period.

## Discussion

All the turtles of the present experiments sooner or later returned to the beach where they had been captured and where most of them successively laid a further clutch of eggs. Since they were displaced at the beginning of the nesting season, were expected to be motivated to complete their nesting cycle with further egg-laying events. Even turtles H and I, that did not make another nesting attempt, returned to the vicinity of home beach. This behaviour does not imply a weak fidelity to the nesting beach, as both turtles were then tracked in following months while migrating to Crete and North Africa (the Authors, data not shown), without any indication that they nested elsewhere. It is therefore likely that these turtles had been displaced after their last egg-laying of the season: if so, they actually displayed quite a strong fidelity to the nesting beach, returning to it even without nesting before starting their migration. Such a strong fidelity is further highlighted by the fact that 6 of the 9 turtles passed close to two known nesting sites present in the area (Fethiye and Dalaman; Fig. 1) without stopping there.

The behaviour of tracked turtles opens the question of whether the original nesting beach was really the goal of displaced turtles, or whether they rather aimed to quickly reach the closest inshore waters, where they could rest and perhaps forage, then reaching the Iztuzu area later on. However, the behaviour of displaced turtles after reaching the coast was not consistent with this second possibility, as they did not stop for a significant time at any coastal site and continued to move at unchanged speed and in a highly oriented manner (Supplementary material Fig. S2). The turtles changed their movement pattern only when they reached the waters off the home beach, where they remained several days before their successive nesting attempt. We therefore conclude that displaced turtles aimed to return to Iztuzu beach, which should then be considered the goal of their homing behaviour. Therefore, they mostly failed to orient immediately towards the nesting beach and their overall homing behaviour generally resembled that shown in previous experiments with turtles displaced offshore, either at short^10^ or long distance^5,14,15,23^.

The orientation data recorded by loggers support this conclusion, showing that the turtles generally oriented in the same direction as the resulting route, and that their initial move not directed towards home was due to a deliberate orientation response, with little or no effect of current drift.

The high temporal resolution of the data recorded by the logger has also revealed the presence of subtle aspects of the turtles’ responses that would have been missed by analysing the satellite tracking data only, like the ability to maintain a given direction at night or the disorientation displayed in the first hour after release by all turtles (Figs. 2, 3). Indications from previous tracking studies have suggested that ocean-moving turtles do not completely rest at night but continue to travel, even if at slower speeds^32,33^. Our findings showing a consistent nighttime orientation provide further support to this view, since it is likely that tracked turtles continued to swim at night while maintaining the same direction. Initial disorientation after a displacement has never been previously noted, likely because it does not produce evident effects on the reconstructed homing routes given the low temporal resolution of Argos tracking. Indeed, in the present case too this initial orientation did not translate into an appreciable worsening of the homing course. The most likely explanation for this initial disorientation is that turtles were affected by the stress due to translocation and release procedures, which may have prevented them to orient in any direction soon after release. A similar stress effect is known to play a role in worsening the orientation of displaced homing pigeons^34^ and likely other birds^35^. On this connection, it has to be noted that, during this short disorientation period, turtles may have not actually moved much away from the release site, only displaying circuitous movements around it or even remaining stationary while orienting in different directions.

The behaviour of most translocated turtles can be interpreted as deriving from a two-phase homing strategy consisting in assuming a consistent orientation for several hours after release (Figs. 2, 3) to reach the continental coastline, and then move along the coast to get back home. Given that the initial orientation was not precisely directed towards destination, turtles made landfall at some distance from home, but were then able to orient homeward afterwards. In doing so, they likely switched to a pilotage mechanism based on locally available information such as coastal landmarks or bathymetric features, even if their final approach to home did not follow a strictly coastal route, and rather involved a crossing of the Iztuzu bay (Supplementary material Fig. S3). A major exception to this pattern is constituted by turtle H, whose initial response led her to reach the coast only after a 2-day oceanic loop. While we do not have logger data for this turtle, we can safely assume, in analogy with the other turtles, that these movements derived from an active orientation of the turtle, that therefore displayed poor homing abilities, without even being helped by coastal cues. The importance of costal cues in turtle navigation has been recently highlighted by satellite tracking data showing that turtles often have difficulty in finding small remote targets, like isolated oceanic islands^36,37^.

Biphasic migratory routes, involving an open-sea leg followed by an extended coastwise segment, have been described in naturally migrating loggerhead and green turtles, especially when aiming to reach large targets like continental coasts^38–43^. This behaviour, which has been deduced only from the features of reconstructed routes, is generally thought to derive from the turtles’ tendency to reduce the time spent in offshore waters, where less foraging opportunities exist. The present findings reveal such a two-phase movement strategy also in turtles subjected to displacement and so aiming to a specific site, in line with hypotheses proposed for adult migrations towards remote islands^44^ and for the special case of homing to the natal site (^45^ and references therein). The fine-scale, water-related orientation recorded in displaced turtles provide strong support to this view, suggesting that navigational constraints may also play a role in determining the indirect routes often observed either during natural migrations or after experimental displacements.

This behaviour indicates that displaced turtles did not possess a precise knowledge of their position at the moment of release, probably as a consequence of their reliance on a coarse-resolution navigational map, that only provided them with a crude estimate of their position with respect to home. This also applies to the two turtles that did not attempt to nest, which first returned to Iztuzu area before starting their post-nesting migration. Other displacement experiments have documented a similar tendency of turtles to maintain a persisting orientation in a given direction weakly related to home, following open-sea release^14,15,23^. In these cases, turtles managed to reach home only after long circuitous paths since they had to perform a more demanding task than the turtles of the present study, having been released far from home and in open ocean without any coastal landmark to help them in correctly orient towards home.

It is unclear why displaced turtles have chosen to consistently orient in a direction not oriented towards home, nor why most of them displayed a similar northeast orientation in the first hours after release. In the days of releases, winds around the release sites were mostly from the west or south, and currents were weak and flowing from northwest or north (data from Mediterranean Forecasting System model), so the turtles’ northeast orientation seems not related to neither winds nor currents. One possible interpretation of this choice could be related to the (unknown) route these turtles followed during their pre-nesting migration towards Iztuzu beach. It is known that most loggerheads nesting in this area migrate towards west or southwest to reach foraging grounds located along the North African shelf or the Greek archipelago, and indeed four of the translocated turtles were then tracked during their successive migration in a generally southwest- or west-ward direction (the Authors, data not shown). If so, it can be hypothesised that the northeast orientation exhibited after release may resemble the one turtles had kept in their pre-breeding journey towards the Iztuzu area some weeks before the experiment. Birds subjected to experimental displacements have often been shown to adopt a fixed orientation not directed towards home after release, especially when unable to navigate and correct for the displacement (e.g.^7,46^.). These bird responses are thought to derive from a sort of revert to their inherited spatio-temporal programme that guides the first migration of their life^47^. In sea turtles, the ontogeny of migration and navigation abilities is much more complex than relying on inherited programmes, as it linked to the prolonged passive transport phase of the first years of life^48^, although it has been suggested that adult turtles too may rely on some form of spatio-temporal programme, possibly determined by their initial migratory experiences, for their long-distance migrations^49^. It is therefore tempting to suggest that also the fixed orientation responses displayed by translocated turtles may derive from their reliance on such a strategy, that may constitute a sort of back-up emergency response^4^, activated when other navigational mechanisms fail, like after a displacement.

The present results reveal that marine turtles subjected to displacements rely on a biphasic homing mechanism, which is in line with the modern general framework of long-distance navigation in migratory animals, that is thought to derive from the sequential reliance of navigational mechanisms acting at different spatial scales^4,18^. Under such scenario, turtles displaced over moderate distances (e.g. < 100 km) like in this case, may be within an area where global scale map-like navigational mechanisms are of little use, because based on crude navigational maps. They will then have to rely on regional-scale system(s) that may not permit an accurate determination of their position with respect to home, producing the indirect homing paths that we observed. While the details of how such a general strategy may apply to turtles are presently unknown (e.g. which are the navigational cues involved, or the extensions of the areas where successive mechanisms start to play a role), interpreting the data available on sea turtle migrations and navigation within this multi-phase framework seems to be the most promising avenue for future studies.

## Materials and methods

### Study site and general procedures

In 2017 and 2018, three displacement experiments (E1, E2 and E3) were performed with a total of nine nesting females (Supplementary material Table S1). Turtles were caught at the beginning of the nesting season (end of May—middle of June) on Iztuzu beach, Dalyan, southwestern Turkey. The area hosts one of the main rookeries of loggerhead turtles in the Mediterranean Sea, with 400–658 nests laid per year^50^. Turtles were approached at the end of the egg-laying process and kept in tanks at the nearby rehabilitation centre for 1–3 days. They were then equipped with satellite transmitters and data loggers and translocated to offshore sites 18, 50 and 56 km from the nesting beach (Fig. 1a,b). In experiments E1 and E2, turtles were embarked on a boat leaving directly from the beach, while the three turtles of experiment E3 were firstly driven to the town of Göcek (Fig. 1a) by van, and then embarked on a boat from the local harbour. The study was authorised by the Turkish Ministry of Environment and Urbanization (General Directorate For Protection Of Natural Assets, Permission 18/11/2015-E.21697-88056259-746.01.02.01/) and by Pamukkale University Animal Experimentation Ethics Committee (permit PAUHDEK-2014/016 number 2014/05, to Prof. Yakup Kaska). The experimental procedures complied with institutional and national relevant guidelines and regulations.

Eight of the translocated turtles were equipped with standard Argos transmitters (model TAM 2638 and TAM 2640, Telonics, Mesa, AZ USA), while turtle H had a GPS transmitter linked to the Iridium satellite network (Telonics model SeaTrkr 4370-4). The unit featured a GPS receiver with a rapid technology called Quick Fix Pseudoranging (QFP) and was programmed to get one position every 30 min to be relayed to the Iridium satellites. Tags with QFP obtain the data necessary to estimate the location in about 3 s, thus work very well for airbreathing marine animals. QFP location accuracy is virtually the same as standard GPS (typically better than 25 m) and varies according to the number and spatial arrangement of GPS satellites visible during each contact^51^.

All turtles additionally carried a multi-sensor data logger (model AGM-D, Technosmart srl, Rome, Italy) that recorded geomagnetic intensity in the three axes with a sampling period of 1 s, along with depth and tri-axial acceleration. The two instruments were glued to the carapace of each turtle using epoxy glue (Dewalt Pure-110 Pro Epoxy, Maryland-USA): the transmitter was placed in the highest part of the carapace to facilitate contacts with the satellites, while the logger was attached 5–10 cm backward, aligned with the longitudinal axis of the turtle. Specific measures performed before the test revealed no magnetic influence of the transmitter on the logger’s magnetic sensor.

Since turtles were translocated well before the end of the nesting season, they were expected to return to Iztuzu beach to complete their egg-laying cycle, so that they could be recaptured at the following nesting attempt to recover the data loggers. Six out of nine turtles were retrieved in this way, with the logger being removed to download the data while the satellite transmitter was left in place. In 2018, we removed also the satellite transmitters from two turtles that homed to Iztuzu after the first displacement in order to reuse the same instrument for the following experiment. It was not possible to obtain blind data because they were obtained from instruments deployed on animals in the field.

### Location data analysis

Data obtained from the satellite transmitters were processed by Argos using Kalman filter and we further discarded Argos locations that were on land and those with an estimated travel speed exceeding a predetermined threshold of 5 km/h^52^. GPS locations were classified into three categories basing on the number of satellites used to calculate locations^51^: (i) Resolved QFP (a minimum of 4 GPS satellite signals), (ii) Resolved QFP—Uncertain (3 satellites), and (iii) Unresolved QFP (< 3 satellites). Locations of the category “Unresolved QFP”, were removed for the analysis and the other ones were subjected to the same filtering process used for Argos locations, after which no location was however removed. Argos routes were also interpolated with a time interval of 1 h, to obtain a closer matching with logger data. Turtle overall homing efficiency was assessed by calculating the track straightness index, expressed as the ratio between the beeline distance from the release site to home and the length of the homing path, which is known to provide a reliable representation of the orientation efficiency of a tracked path^53^. Homing path length was calculated until the first location obtained within 3 km of Iztuzu beach. For turtle H, that did not get back to the beach, the point where she reached the coast was considered as final point (see also Sect. 3.3), while for turtle F, whose tracking was interrupted before reaching the home beach, path length was calculated by adding a final segment joining the last location obtained to the home beach.

### Heading data analysis

The heading of the turtles was estimated from the logger’s 3-axis accelerometer and magnetometer measurements through custom-made scripts in Matlab R2016b. The magnetometer was first calibrated with a new calibration method based solely on the measurements recorded during the turtle’s own movements, during which it actively and richly oriented its body in 3D. In this way, magnetometer calibration is not affected by any metallic and magnetic disturbance existing on the operation site before release and takes then into account the actual magnetic environment that the turtle encounters during its journey. A sphere was fitted on the 3D point cloud deriving from the raw magnetometer data and the sphere centre and radius was assessed using the least-squares method. The coordinates of the sphere centre determined the magnetometer biases along x, y, and z axes. A gentle Savitzky-Golay smoothing filter (order: 2, window: 3^54^) was applied to the magnetometer data to remove high-frequency noise before subtracting the biases. The debiased magnetometer data were then normalized by the radius of the fitted sphere to compute the calibrated magnetometer data along the three axes. During this process, it was noted that the data recorded by turtle A’s logger included clearly shifted magnetometer recordings leading to greatly distorted spheres. It was therefore decided to limit the analysis of these data to the recordings obtained in the first 5.5 h, that did not present such biased recordings.

Turtle pitch and roll along its path were then estimated starting from the acceleration components recorded by the loggers along the homing path by using classical static attitude equations that are based on basic trigonometry (see^55^). Pitch and roll values were then filtered by a gentle Savitzky-Golay smoothing filter (order: 2, window: 3) to remove high-frequency noise. Finally, the heading of the turtle was assessed by referring to the horizontal magnetometer component compensated for the turtle’s pitch and roll using the formulas:

$$ \begin{gathered} MAGX \, = \, magx*cos\left( {Pitch} \right) \, + \, magy*sin\left( {Roll} \right)*sin\left( {Pitch} \right) \, + \, magz*cos\left( {Roll} \right)*sin\left( {Pitch} \right) \hfill \\ MAGY \, = \, magy*cos\left( {Roll} \right) \, - \, magz*sin\left( {Roll} \right) \hfill \\ \end{gathered} $$

where magx, magy and magz are the values of the magnetic components recorded by the logger, Pitch and Roll are the values previously calculated.

The heading of turtle could then be computed referring to the horizontal tilt-compensated magnetometer component along its path with the formula:

$$ Turtle\_Heading \, = \, arctan\left( {MAGX/MAGY} \right). $$

A fixed value of 4.8° was then subtracted to the magnetic headings calculated in this way to account for magnetic declination in the area, as determined by the NOAA’s magnetic field calculator (https://www.ngdc.noaa.gov/geomag/calculators/magcalc.shtml) according to the International Geomagnetic Reference Field model.

Heading data were processed with standard circular statistics methods^56^, calculating mean vector direction and length for successive samples of magnetic headings. Mean vector length is a measure of dispersion of a circular data sample and thus provides indications on the turtle tendency to maintain a given direction, with values close to 0 representing a uniformly scattered distribution and values close to 1 indicating a fully concentrated distribution. The homeward component of these vectors was computed as *Hc* = R*cos(a-b), with R = mean vector length, a = orientation of the turtles’ mean vector, b = direction of the home beach^56^. Diel differences in turtle orientation were studied referring to data obtained on the day of releases E1 and E2, when all turtles were still in open sea, at least 15 km away from the nearest coast. For this we calculated mean vectors in two successive 60-min periods that were completely diurnal or nocturnal, one ending 10 min before nautical dusk and the other starting 10 min after nautical twilight of the day of release. Differences in the resulting mean vector distributions were tested with the Hotelling one-sample test^56^.

## Supplementary information

Supplementary Information.
