# Supplementary material for: A biphasic navigational strategy in loggerhead sea turtles
Source: Sci Rep. 2020 Oct 22;10:18130. doi: 10.1038/s41598-020-75183-6 (PMC7581759; doi:10.1038/s41598-020-75183-6)
Supplement: Supplementary file 1 — Supplementary Information. [file 41598_2020_75183_MOESM1_ESM.pdf]

Supplementary material

A biphasic navigational strategy in loggerhead sea turtles

Paolo Luschi[a]*, Dogan Sözbilen[b], Giulia Cerritelli[a], Franck Ruffier[c], Eyup Baskale[b], Paolo Casale[a]

[a] *Department of Biology, University of Pisa, Italy*

[b] *Department of Veterinary, Acıpayam Vocational School, Pamukkale University, Denizli, Turkey*

[c] *Aix Marseille University, CNRS, ISM, Marseille, France*

*paolo.luschi@unipi.it

Table S1 *Details of experimental turtles and homing performances. CCL: curved carapace length. SI: straightness index (see text)*

Turtle	CCL (cm)	Release date/time	End of homing trip	Homing path length (km)	SI	Data logger retrieval date/time
**Exp. E1**						
A	69.7	28/06/2017 18:37	29/06/2017 10:29	25.24	0.73	10/07/2017 23:50
B	79.5	29/06/2017 18:38	29/06/2017 18:19	35.58	0.52	-
**Exp. E2**						
C	81.5	25/05/2018 11:35	27/05/2018 04:03	64.85	0.76	07/06/2018 01:05
D	74.3	25/05/2018 11:40	27/05/2018 11:42	90.14	0.54	06/06/2018 21:35
E	80	25/05/2018 11:36	30/05/2018 12:00	140.66	0.35	08/06/2018 22:12
F	73.5	25/05/2018 11:33	Unknown	252.68[a]	0.22	22/06/2018 02:55
**Exp. E3**						
G	84	13/06/2018 11:11	18/06/2018 09:00	173.24	0.32	25/06/2018 22:45
H	74	13/06/2018 11:12	-	117.77[b]	0.47	-
I	69	13/06/2018 11:11	25/06/2018 09:43	119.73	0.46	-

[a] Calculated by adding a final segment from the last Argos location to home

[b] Considered the location where she reached the coast as endpoint

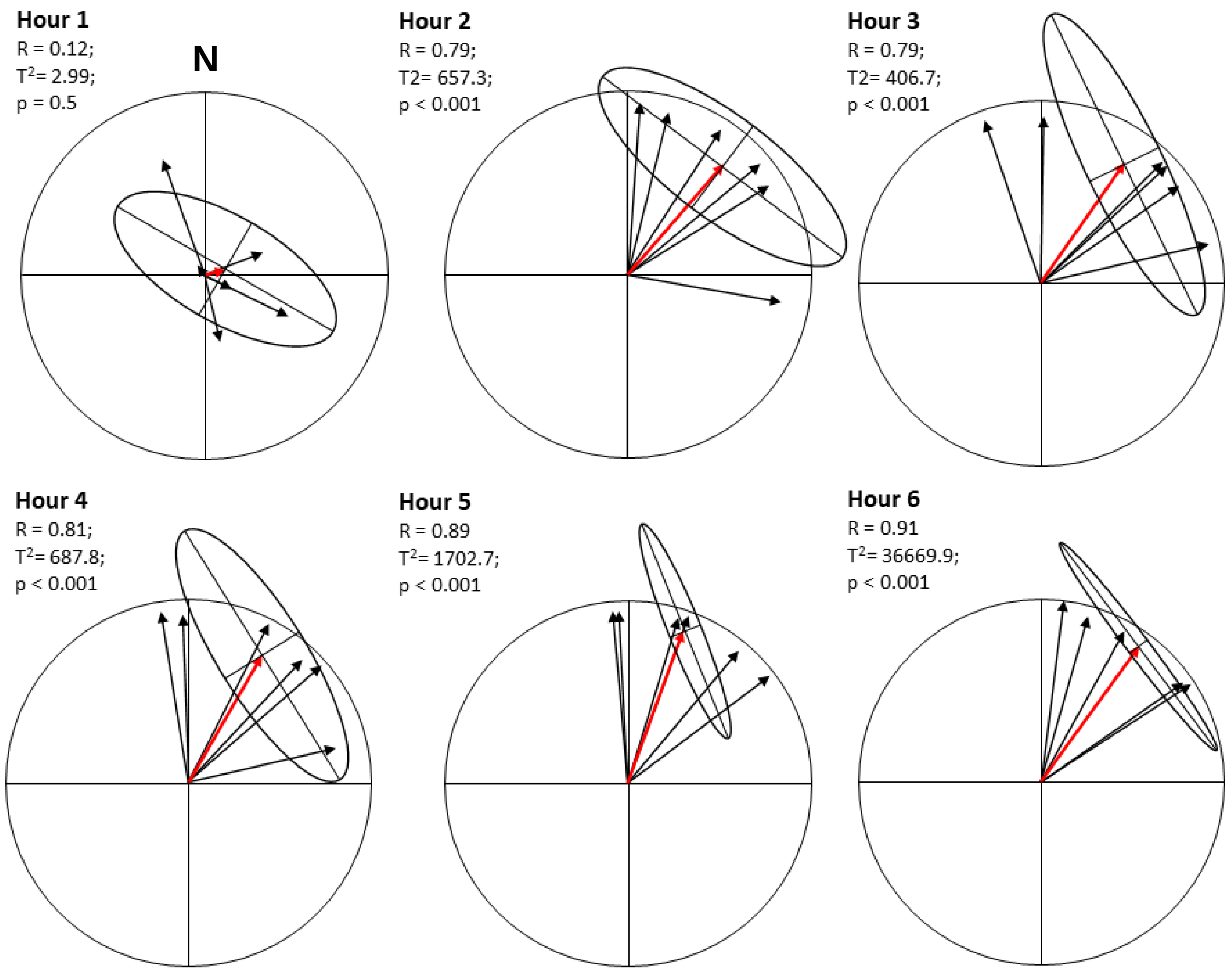

Figure S1. *Hourly mean vector distribution in the 6 monitored turtles in the first 6 hours after release and related confidence ellipses. In each diagram, black arrows represent the mean vectors calculated from the hourly heading data (n=3600 in all cases) of each turtle, the red arrow represents the second-order grand mean vector of the distribution, whose length (R) is given, together with the results of the corresponding Hotelling test.*

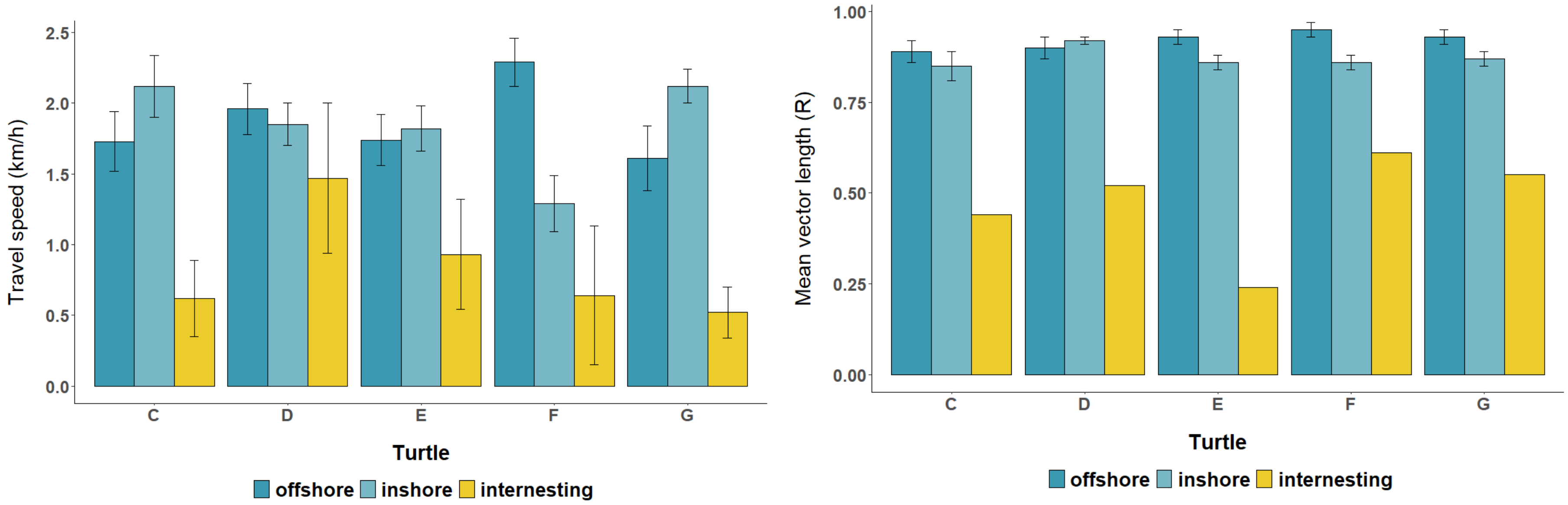

Figure S2. *Travel speeds and orientation of the five displaced turtles that moved along the coast, during the initial offshore leg, the subsequent inshore one, and after reaching the home beach waters (internesting). Left panel: mean (±SEM) speed recorded between successive Argos locations. Right panel: mean (±SEM) vector length (R) of hourly samples of magnetic headings (see Fig. 2 for further explanations); internesting values show the mean vector length calculated in the first hour after homing was completed.*

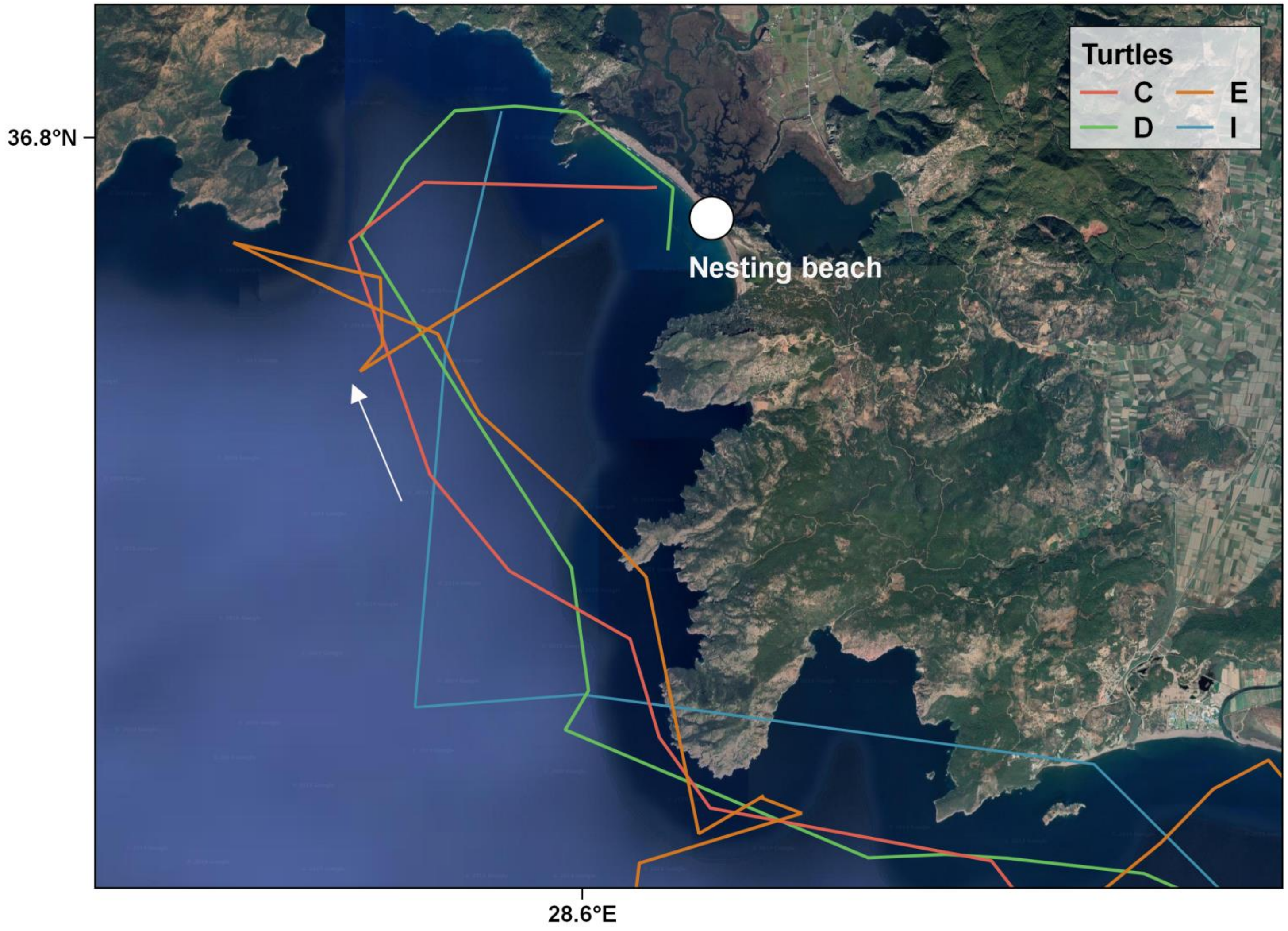

Figure S3 *Close-up of the final approach to Iztuzu beach (white dot) of turtles released in experiments E2 and E3. The map was created on QGIS 3.12.2 (https://www.qgis.org). Map data: Google.*
